# Atypical Scene‐Selectivity in the Retrosplenial Complex in Individuals With Autism Spectrum Disorder

**DOI:** 10.1002/aur.70079

**Published:** 2025-06-25

**Authors:** Andrew S. Persichetti, Taylor L. Li, W. Dale Stevens, Alex Martin, Adrian W. Gilmore

**Affiliations:** ^1^ Section on Cognitive Neuropsychology, Laboratory of Brain and Cognition National Institute of Mental Health, National Institutes of Health Bethesda Maryland USA; ^2^ Department of Psychology York University Toronto Canada; ^3^ Department of Psychological and Brain Sciences University of Delaware Newark Delaware USA

**Keywords:** autism spectrum disorder, functional MRI, navigation, scene perception, spatial cognition

## Abstract

A small behavioral literature on individuals with autism spectrum disorder (ASD) has shown that they can be impaired when navigating using map‐based strategies (i.e., memory‐guided navigation), but not during visually‐guided navigation. Meanwhile, there is neuroimaging evidence in typically developing (TD) individuals demonstrating that the retrosplenial complex (RSC) is part of a memory‐guided navigation system, while the occipital place area (OPA) is part of a visually‐guided navigation system. A key identifying feature of the RSC and OPA is that they respond significantly more to pictures of places compared to faces or objects—i.e., they demonstrate scene‐selectivity. Therefore, we predicted that scene‐selectivity would be weaker in the RSC of individuals with ASD compared to a TD control group, while the OPA would not show such a difference between the groups. We used functional MRI to scan groups of ASD individuals and matched TD individuals while they viewed pictures of places and faces and performed a one‐back task. As predicted, scene‐selectivity was significantly lower in the RSC, but not OPA, in the ASD group compared to the TD group. These results suggest that impaired memory‐guided navigation in individuals with ASD may, in part, be due to atypical functioning in the RSC.

**Summary**

- The retrosplenial complex (RSC), a cortical region that is part of a neural system that supports our ability to form map‐like mental representations of the environment and use them to navigate (i.e., memory‐guided navigation), exhibits atypical responses to images of places in individuals with autism spectrum disorder (ASD).
- These results are a first step toward understanding the neural mechanisms responsible for understudied behavioral impairments in memory‐guided navigation in individuals with ASD.

## 1 Introduction

The core impairments that define autism spectrum disorder (ASD) are related to social communication and restricted and repetitive behaviors (Marco et al. 2011; APA DSM‐V 2013). However, researchers have highlighted other cognitive domains that may differ in individuals with ASD, such as autobiographical memory retrieval (Kanner 1943; Cooper and Simons 2019; Agron et al. 2024) and map‐based (also referred to as “memory‐guided” or “allocentric”) navigation—i.e., the ability to form map‐like mental representations in memory and use them to navigate to out‐of‐sight places in the broader environment (Lind et al. 2013, 2014; Ring et al. 2018; Yang et al. 2021; for a recent review relating memory and navigation in ASD, see Agron et al. 2024). To date, however, reports of atypical navigation behaviors in ASD have not been accompanied by neuroimaging data that might speak to potential underlying mechanisms.

Several studies using functional MRI (fMRI) have demonstrated that the retrosplenial complex (RSC) and occipital place area (OPA) both respond significantly more to images of places (also called “scenes”) than to images of faces and objects, thus earning them the distinction of being scene‐selective regions of human cortex (Maguire 2001; Dilks et al. 2013). Although other regions, such as the parahippocampal place area (PPA) are also scene‐selective, RSC and OPA are particularly useful targets because they are parts of dissociable neocortical systems that are involved in memory‐guided navigation and visually‐guided navigation, respectively (see Dilks et al. 2022 for review). Since the RSC is part of a neural system that is responsible for the type of memory‐guided navigation that is impaired in individuals with ASD, we used fMRI to ask whether processing of places or scenes might be atypical in a group of ASD participants. Specifically, based on the observations of memory‐based navigation difficulties in ASD, we predicted that the RSC in individuals with ASD would show significantly weaker scene‐selectivity when compared to a TD control group. In contrast, the OPA was not predicted to show between‐groups differences, because existing evidence does not suggest differences in visually‐guided navigation behavior in individuals with ASD. Finally, the prediction for a third scene‐selective cortical region—the parahippocampal place area (PPA)—was less clear since its exact role in navigation is not as clearly specified (with putative roles ranging from landmark recognition to scene categorization; Epstein 2008; Dilks et al. 2022). Thus, the current experiment is a direct test of whether a key cortical region involved in memory‐guided navigation (RSC) is specifically impaired—compared to a cortical region involved in visually‐guided navigation (OPA) – in a group of individuals with ASD, while also investigating responses across the scene processing system more generally in these individuals.

## 2 Materials and Methods

### 2.1 Participants

Twenty individuals [age, mean (SD) = 19.48 (2.9) years] who met the DSM‐V criteria for ASD (APA DSM‐V 2013), as assessed by a trained clinician, were recruited for this experiment. All were high functioning and without intellectual disability. In addition, 19 individuals with no history of psychiatric or neurological disorders [mean (SD) age = 21.01 (4.2) years] served as the TD control group (Some of the data from the TD group were previously described in Stevens et al. 2015, 2017). There were no significant differences between the two groups in age (*t*_(37)_ = 1.31, *p* = 0.20) or overall IQ (Full‐score IQ, mean (SD): ASD: 115.4 (12); TD: 119.3 (11.6), *t*_(37)_ = 1.04 *p* = 0.31), which was measured using the Wechsler Abbreviated Scale of Intelligence (Wechsler 1999) within 1 year of the scanning session in all participants. All participants from both groups were males with normal or corrected‐to‐normal visual acuity. Informed assent and consent were obtained from all participants and/or their parent/guardian when appropriate, and all methods used in this study followed ethical guidelines and regulations in accordance with a National Institutes of Health (NIH) Institutional Review Board.

### 2.2 MRI Data Acquisition and Experimental Task Procedure

Scanning was completed on a General Electric Signa 3 Tesla scanner (GE Healthcare) with an 8‐channel receive‐only head coil at the NIH Clinical Center NMR Research Facility. For each participant, T2*‐weighted blood oxygen level‐dependent (BOLD) images covering the whole brain were acquired using a gradient echo single‐shot echo planar imaging sequence (repetition time = 2000 ms, echo time = 27 ms, flip angle = 77°, 41 axial contiguous interleaved slices per volume, 3.0‐mm slice thickness, field of view = 216 mm, 72 × 64 acquisition matrix, single‐voxel volume = 3.0 mm isotropic). In addition to the functional images, a high‐resolution T1‐weighted anatomical image (magnetization‐prepared rapid acquisition with gradient echo—MPRAGE) was obtained (124 axial slices, 1.2 mm^3^ single‐voxel volume, 256 × 256 acquisition matrix, field of view = 24 cm).

**FIGURE 1:**
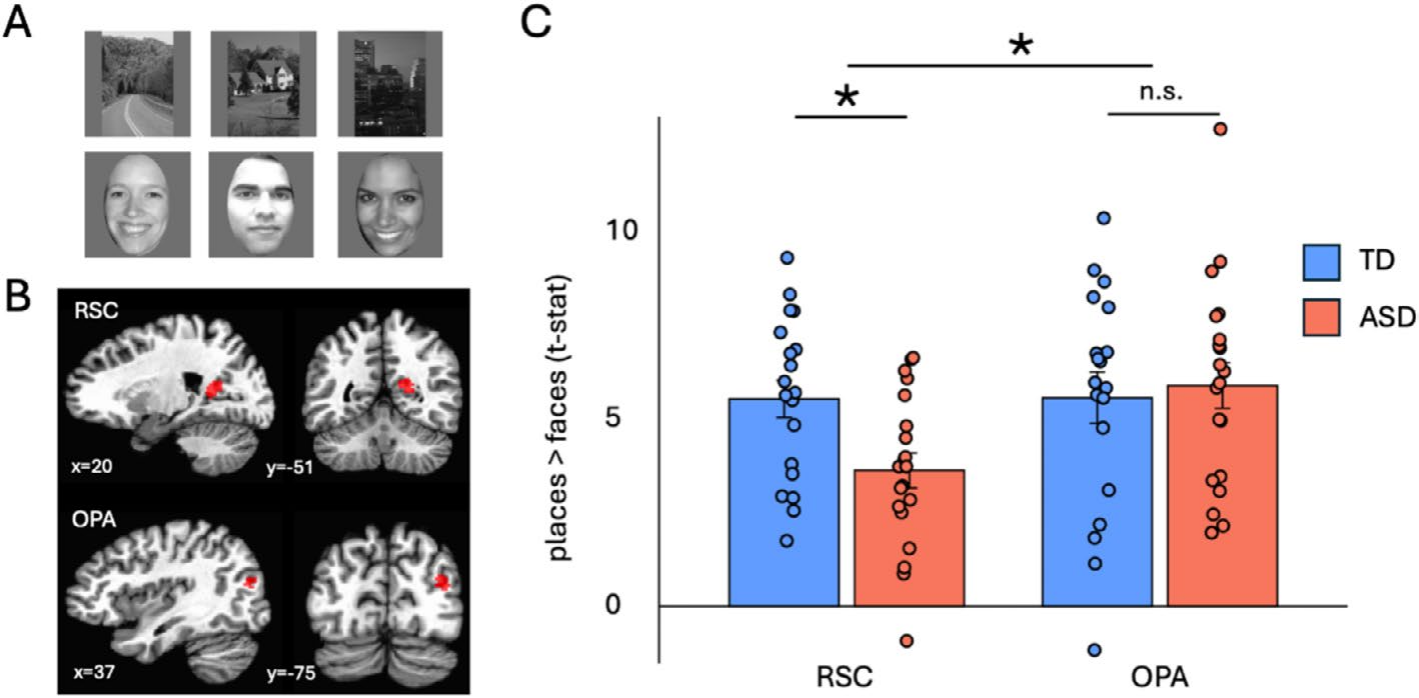
(A) Example images of places and faces. (B) An example of the functionally defined RSC and OPA. The size and location of both regions of interest were very similar between the ASD and TD groups. (C) A mixed‐effects ANOVA showed that scene selectivity (places>faces) was weaker in the RSC, but not in the OPA, of the ASD group compared to the TD group.

During scanning, all participants completed six runs of a multicategory functional localizer with a block‐design task. Each run lasted 7 m 18 s (219 brain volumes), and independent measures of nuisance physiological variables (cardiac and respiration) were recorded during all scans for later removal. Each run comprised 14 task blocks (20 s each) interleaved with 13 fixation blocks (10 s each), with an additional fixation block at the beginning (18 s). There were 14 different categories of stimuli used in the original study (for more details, see Stevens et al. 2015). However, we focused on places and faces (elliptically cropped to exclude hair and clothes) for the primary contrast in this report (see Figure 1A), with a supplemental analysis using places, tools, abstract objects, and non‐manipulable objects. All stimuli were grayscale images of the same size (600 × 600 pixels) and were projected onto a screen behind the scanner and viewed via a mirror mounted to the head coil. Each block contained 20 pictures from the same category, with each picture presented for 300 ms, followed by a 500‐ms interstimulus interval. To ensure attention to each stimulus, participants performed a one‐back task, responding by pressing a button with their left index finger every time the same picture was presented twice in a row (this happened 1–2 times per block).

All data were preprocessed using the AFNI software package (Cox 1996). First, the initial four TRs from each EPI scan were removed using 3dTcat to allow for T1 equilibration. Next, 3dDespike was used to bound outlying time points in each voxel within four standard deviations of the time series mean and 3dTshift was used to adjust for slice acquisition time within each volume (to *t* = 0). Each volume of the scan series was then aligned to the first retained volume of the scan using 3dvolreg. Finally, all scans were spatially blurred by a 6‐mm Gaussian kernel (full width at half maximum) and divided by the mean of the voxelwise time series to yield units of percent signal change. To ensure that the fMRI data quality from both groups was matched, we computed the temporal signal‐to‐noise‐ratio (tSNR) across the whole brain as well as a summary of in‐scanner head motion using the @1dDiffMag program in AFNI. The groups did not differ in tSNR (*t*_(37)_ = 1.62, *p* = 0.11) or in‐scanner head motion (*t*_(37)_ = 1.72, *p* = 0.10).

After preprocessing, we used data from two of the task runs to functionally define the RSC and OPA bilaterally as regions that responded significantly more to pictures of places than faces (i.e., that demonstrated scene‐selectivity—Figure 1B). This was done separately in each participant. We then extracted the average response to each category during the remaining four runs from each region of interest (ROI) for further analysis (see Figure S1 for responses to places and faces plotted separately in each ROI). Thus, the data used to functionally define each ROI and the data used for subsequent analyses are independent of one another and provide an internal replication of scene‐selectivity within each ROI of each participant. Although the right RSC and OPA were defined in every participant, we could not define the left RSC or OPA in every participant: in the TD group, we defined 18/19 left RSC's and 16/19 left OPA's, while in the ASD group, we defined 15/20 left RSC's and 19/20 left OPA's. This relative difficulty in functionally defining navigationally relevant regions of interest is consistent with evidence that navigational functions are biased to the right hemisphere of the brain (see Maguire 2001 for review). Therefore, we focused our analyses on the right hemisphere only (see Figure S2 for analysis of the ROIs in the left hemisphere). For each participant, a third scene selective cortical region—the parahippocampal place area (PPA) was defined for supplementary analysis, as was the hippocampus (defined anatomically using Freesurfer).

## 3 Results

First, the size and location of the RSC and OPA were compared between the TD and ASD participants. The sizes of each ROI did not significantly differ between the groups: the average number of 3mm^3^ voxels (in each participant's native space) between the TD and ASD groups in the RSC was 9.58 and 11.05, respectively (*t*_(37)_ = 0.68, *p* = 0.50, Cohen's *d* = 0.22), while the number of voxels in the OPA was 14.82 and 14.5, respectively (*t*_(37)_ = −0.08, *p* = 0.94, Cohen's *d* = 0.03). Additionally, the Euclidean distances between the centers of mass for the average RSC and OPA across groups were less than one voxel's width away from one another—1.75 mm and 2.22 mm, respectively.

Next, to test our hypothesis that scene‐selectivity in ASD individuals is atypical in the RSC, but not the OPA, we conducted a mixed‐effects ANOVA with one between subjects factor (Group: TD, ASD) and one within‐subjects factor (ROI: RSC, OPA). As predicted, we found a significant Group × ROI interaction (*F*_(1,37)_ = 5.08, *p* = 0.03, η_p_^2^ = 0.12) and independent‐samples *t*‐tests confirmed that scene‐selectivity in the RSC of the ASD group was weaker than the TD group (*t*_(37)_ = 2.81, *p* < 0.01, Cohen's *d* = 0.90), whereas there was no difference in scene‐selectivity in the OPA between the groups (*t*_(37)_ = 0.36, *p* = 0.72, Cohen's *d* = 0.12—Figure 1C). A reasonable concern using this approach is that the use of faces as a comparison may be distorting the current results, given that social deficits are a core impairment associated with ASD. Two additional analyses suggest that this is not the case. First, when plotting responses to places and faces in RSC and OPA separately against a fixation baseline, there was no evidence that an atypical response to faces alone was driving scene selectivity in either ROI (Figure S1). In addition, we also examined scene‐selectivity in RSC using various types of objects as a baseline (tools, non‐manipulable objects, and abstract objects). In all cases, scene selectivity was weaker in the RSC of the ASD group (all *t*'s > 2.25, all *p*'s < 0.05; Figure S3). Finally, to further contextualize the differences observed between groups in RSC but not in OPA, we expanded our analyses to two other regions associated with scene processing: the parahippocampal place area (PPA) and the hippocampus. Independent‐samples *t*‐tests showed that there was not a significant difference in scene‐selectivity between the groups in either the PPA (*t*_(37)_ = 0.67, *p* = 0.51, Cohen's *d* = 0.22) or the hippocampus (*t*_(37)_ = −1.07, *p* = 0.29, Cohen's *d* = 0.35). Across all analyses, therefore, the RSC was unique in differing in scene‐selectivity between the ASD and TD groups.

## 4 Discussion

In this report, we tested the hypothesis that impairments in memory‐guided navigation in individuals with ASD may be due to atypical functioning in the RSC—a key node of the neural system thought to underly memory‐guided navigation in humans. Consistent with our predictions, we found that scene‐selectivity was significantly lower in the RSC, but not in the OPA (which is instead involved in the distinct process of visually‐guided navigation) in a group of individuals with ASD compared to a tightly matched TD control group. These results converge with recent work that found resting‐state functional connectivity within a brain network that supports memory‐guided navigation, and that included the RSC, to differ between groups of ASD and TD individuals (Persichetti et al. 2024). The current results are also consistent with a recent proposal that scene construction—a process by which we mentally generate and maintain a coherent representation of spatial contexts, or “scenes,” using information from memory—may be disrupted in ASD (Agron et al. 2024), as the RSC is strongly associated with this process (Hassabis et al. 2007; Vann et al. 2009). Scene construction differences therefore offer a parsimonious explanation for differences relating to memory‐based navigation and autobiographical memory in ASD, thus the presently reported results are a first step toward understanding the neural mechanisms associated with multiple cognitive differences between ASD and neurotypical individuals. Taken together, the results reported here address relatively understudied impairments related to memory‐guided navigation (and scene construction more generally) in ASD and thus may lead to a better understanding of the heterogeneous phenotypes observed in individuals with ASD. In this regard, it should be noted that our participants were all high‐functioning males, as typically found in studies of ASD. Therefore, it will now be critical to further study the broader memory‐guided navigation system, including the RSC, using neuroimaging methods and more focused navigation‐related paradigms across different environmental contexts, and in larger and more diverse populations of individuals with ASD.

## Author Contributions

A.S.P., A.W.G., W.D.S. and A.M. designed research; A.S.P., T.L.L., A.W.G., and W.D.S. performed research and analyzed data; A.S.P., A.W.G., and A.M. wrote the paper.

## Conflicts of Interest

The authors declare no conflicts of interest.

## Supporting information


**Figure S1.** Responses to places and faces against a fixation baseline, plotted separately in each ROI. Scene‐selectivity is a composite measure that summarizes differences in how a brain region responds to scenes (or ‘places’) as compared to other categories. However, as individuals with ASD have known differences related to face processing, it is worth examining the responses to places and faces separately to better understand whether the scene selectivity difference observed in the RSC of individuals with ASD can be attributed to atypical responses to places, faces, or both. Independent‐samples *t*‐tests showed a marginal decrease in the response of the RSC to places in the ASD group compared to the TD group (*t*_(37)_ = 1.65, *p* = 0.11) and a marginal increase in the response to faces in the ASD group compared to the TD group (*t*_(37)_ = −1.76, *p* = 0.09). Therefore, reduced scene selectivity appears to be due to a combination of atypical responses to both places and faces in the canonically scene‐sensitive RSC. Additionally, there were no significant differences in responses to places and faces between the groups in the OPA, PPA, or Hippocampus (all *t*‐values < 1.49, all *p*‐values > 0.15).
**Figure S2.** Analysis of the scene‐selective ROIs in the left hemisphere. In addition to analyzing responses in the right hemisphere ROIs in the main article, we also looked at scene‐selectivity differences across groups in the left hemisphere (although these were less reliably identified in each participant, see Methods). Independent‐samples *t*‐tests showed no significant difference in scene‐selectivity across the groups in any of the left‐hemisphere ROIs (RSC: *t*_(31)_ = 1.24, *p* = 0.23; OPA: *t*_(32)_ = 0.73, *p* = 0.47; PPA: *t*_(35)_ = 0.89, *p* = 0.38; Hippocampus: *t*_(37)_ = 0.50, *p* = 0.63).
**Figure S3.** Group differences in scene selectivity in the RSC using different baselines. Specifically, we plotted scene selectivity as a contrast between the response to images of places (P) compared to faces (F—i.e., the main analysis), tools (T), non‐manipulable objects (NO), and abstract objects (AO). In all cases, scene selectivity was weaker in the RSC of the ASD group (all *t*’s > 2.25, all *p*’s < 0.05).

## Data Availability

The data that support the findings of this study are available from the corresponding author upon reasonable request.
