# Supplementary material for: Atypical Scene‐Selectivity in the Retrosplenial Complex in Individuals With Autism Spectrum Disorder
Source: Autism Res. 2025 Jun 25;18(8):1563–7. doi: 10.1002/aur.70079 (PMC12257949; doi:10.1002/aur.70079)
Supplement: Supplementary file 1 — Figure S1. Responses to places and faces against a fixation baseline, plotted separately in each ROI. Scene‐selectivity is a composite measure that summarizes differences in how a brain region responds to scenes (or ‘places’) as compared to other categories. However, as individuals with ASD have known differences related to face processing, it is worth examining the responses to places and faces separately to better understand whether the scene selectivity difference observed in the RSC of individuals with ASD can be attributed to atypical responses to places, faces, or both. Independent‐samples t‐tests showed a marginal decrease in the response of the RSC to places in the ASD group compared to the TD group (t (37) = 1.65, p = 0.11) and a marginal increase in the response to faces in the ASD group compared to the TD group (t (37) = −1.76, p = 0.09). Therefore, reduced scene selectivity appears to be due to a combination of atypical responses to both places and faces in the canonically scene‐sensitive RSC. Additionally, there were no significant differences in responses to places and faces between the groups in the OPA, PPA, or Hippocampus (all t‐values < 1.49, all p‐values > 0.15). Figure S2. Analysis of the scene‐selective ROIs in the left hemisphere. In addition to analyzing responses in the right hemisphere ROIs in the main article, we also looked at scene‐selectivity differences across groups in the left hemisphere (although these were less reliably identified in each participant, see Methods). Independent‐samples t‐tests showed no significant difference in scene‐selectivity across the groups in any of the left‐hemisphere ROIs (RSC: t (31) = 1.24, p = 0.23; OPA: t (32) = 0.73, p = 0.47; PPA: t (35) = 0.89, p = 0.38; Hippocampus: t (37) = 0.50, p = 0.63). Figure S3. Group differences in scene selectivity in the RSC using different baselines. Specifically, we plotted scene selectivity as a contrast between the response to images of places (P) compared to faces (F— [file AUR-18-1563-s001.docx]

**Supplementary information**


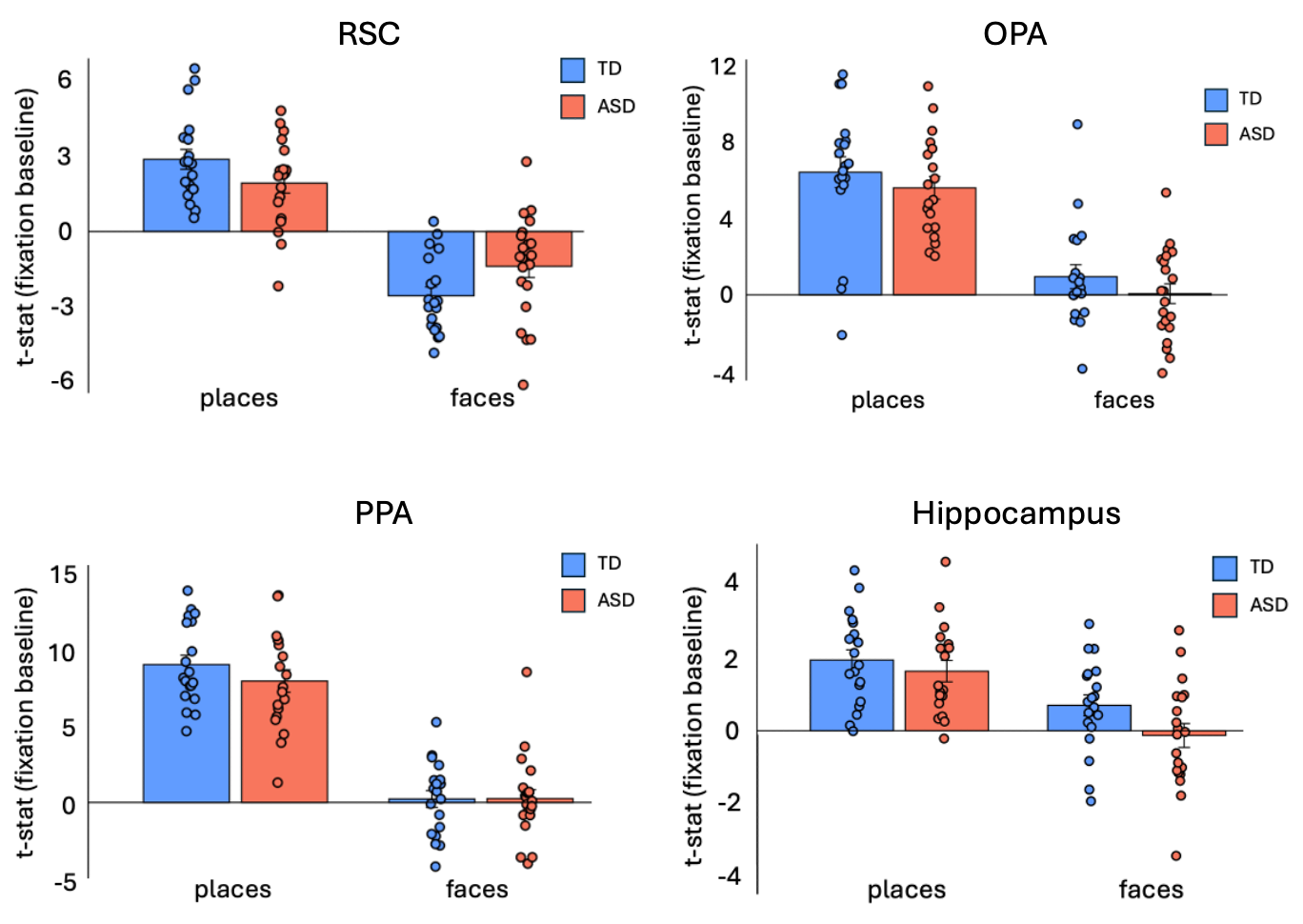


**Supplemental Figure 1**. Responses to places and faces against a fixation baseline, plotted separately in each ROI. Scene-selectivity is a composite measure that summarizes differences in how a brain region responds to scenes (or ‘places’) as compared to other categories. However, as individuals with ASD have known differences related to face processing, it is worth examining the responses to places and faces separately to better understand whether the scene selectivity difference observed in the RSC of individuals with ASD can be attributed to atypical responses to places, faces, or both. Independent-samples t-tests showed a marginal decrease in the response of the RSC to places in the ASD group compared to the TD group (t_(37)_ = 1.65, p = 0.11) and a marginal increase in the response to faces in the ASD group compared to the TD group (t_(37)_ = -1.76, p = 0.09). Therefore, reduced scene selectivity appears to be due to a combination of atypical responses to both places and faces in the canonically scene-sensitive RSC. Additionally, there were no significant differences in responses to places and faces between the groups in the OPA, PPA, or Hippocampus (all t-values < 1.49, all p-values > 0.15).


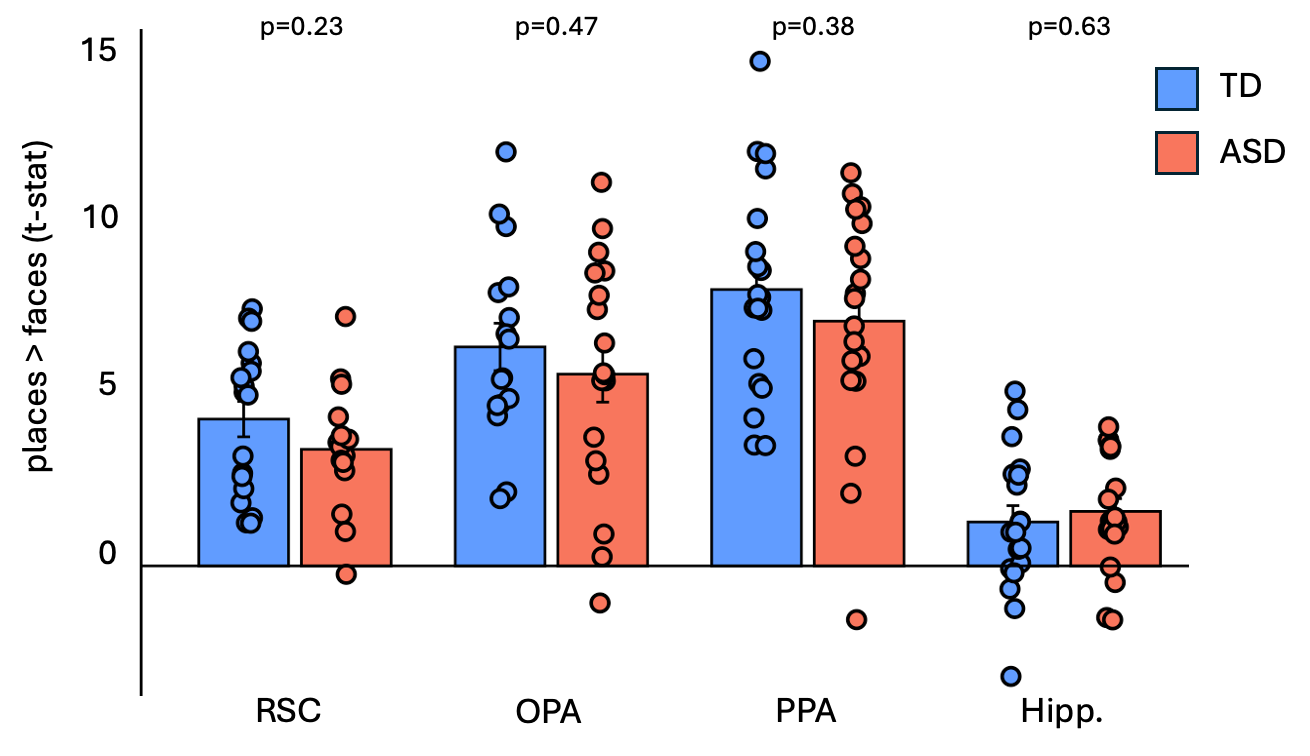


**Supplemental Figure 2.** Analysis of the scene-selective ROIs in the left hemisphere. In addition to analyzing responses in the right hemisphere ROIs in the main article, we also looked at scene-selectivity differences across groups in the left hemisphere (although these were less reliably identified in each participant, see Methods). Independent-samples t-tests showed no significant difference in scene-selectivity across the groups in any of the left-hemisphere ROIs (RSC: t_(31)_ = 1.24, p = 0.23; OPA: t_(32)_ = 0.73, p = 0.47; PPA: t_(35)_ = 0.89, p = 0.38; Hippocampus: t_(37)_ = 0.50, p = 0.63).


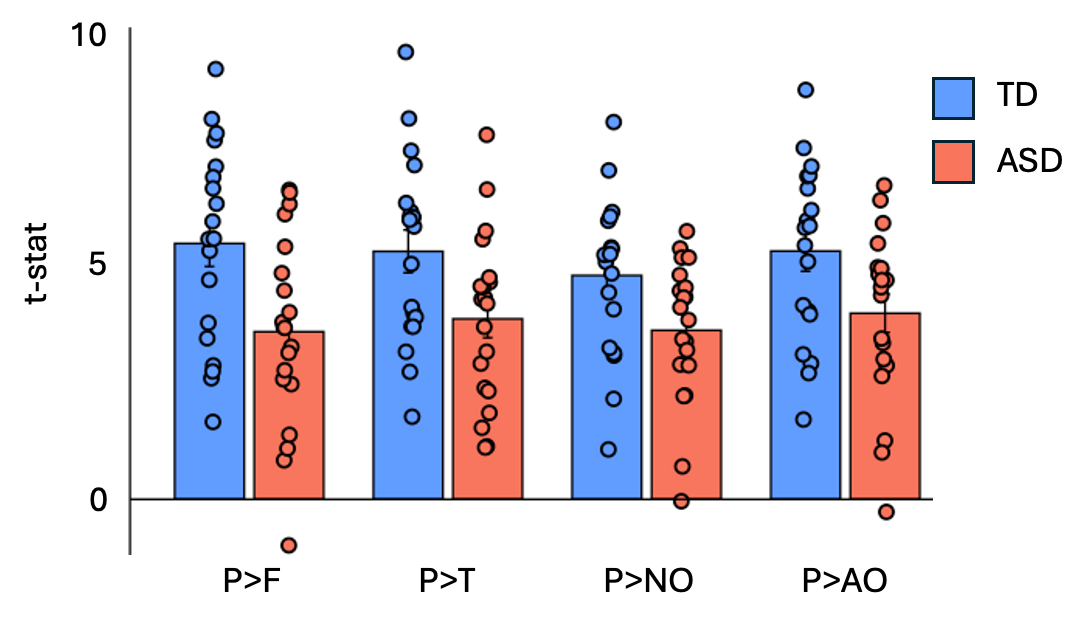


**Supplemental Figure 3.** Group differences in scene selectivity in the RSC using different baselines. Specifically, we plotted scene selectivity as a contrast between the response to images of places (P) compared to faces (F – i.e., the main analysis), tools (T), non-manipulable objects (NO), and abstract objects (AO). In all cases, scene selectivity was weaker in the RSC of the ASD group (all t’s > 2.25, all p’s < 0.05).
